# Clinical Features and Multiplatform Molecular Analysis Assist in Understanding Patient Response to Anti-PD-1/PD-L1 in Renal Cell Carcinoma

**DOI:** 10.3390/cancers13061475

**Published:** 2021-03-23

**Authors:** Eileen Shiuan, Anupama Reddy, Stephanie O. Dudzinski, Aaron R. Lim, Ayaka Sugiura, Rachel Hongo, Kirsten Young, Xian-De Liu, Christof C. Smith, Jamye O’Neal, Kimberly B. Dahlman, Renee McAlister, Beiru Chen, Kristen Ruma, Nathan Roscoe, Jehovana Bender, Joolz Ward, Ju Young Kim, Christine Vaupel, Jennifer Bordeaux, Shridar Ganesan, Tina M. Mayer, Gregory M. Riedlinger, Benjamin G. Vincent, Nancy B. Davis, Scott M. Haake, Jeffrey C. Rathmell, Eric Jonasch, Brian I. Rini, W. Kimryn Rathmell, Kathryn E. Beckermann

**Affiliations:** 1 Medical Scientist Training Program, Vanderbilt University, Nashville, TN 37232, USA; eileen.f.shiuan@vanderbilt.edu (E.S.); stephanie.o.dudzinski@vanderbilt.edu (S.O.D.); aaron.lim@vanderbilt.edu (A.R.L.); ayaka.sugiura@vanderbilt.edu (A.S.); 2 Program in Cancer Biology, Vanderbilt University, Nashville, TN 37232, USA; 3 Prism Bioanalytics, North Carolina Biotechnology Center, Morrisville, NC 27560, USA; areddy@prismbioanalytics.com; 4 Department of Biomedical Engineering, Vanderbilt University, Nashville, TN 37232, USA; 5 Department of Pathology, Microbiology, and Immunology, Vanderbilt University, Nashville, TN 37232, USA; jeff.rathmell@vumc.org; 6 Division of Hematology/Oncology, Department of Medicine, Vanderbilt University Medical Center, Nashville, TN 37232, USA; rachel.hongo@vumc.org (R.H.); jamye.oneal@vumc.org (J.O.); kim.dahlman@vumc.org (K.B.D.); renee.k.mcalister@vumc.org (R.M.); nancy.davis@vumc.org (N.B.D.); scott.haake@vumc.org (S.M.H.); brian.rini@vumc.org (B.I.R.); kimryn.rathmell@vumc.org (W.K.R.); 7 Health Science Center, College of Medicine, The University of Tennessee, Memphis, TN 38163, USA; kyoung50@uthsc.edu; 8 Department of Genitourinary Medical Oncology, The University of Texas MD Anderson Cancer Center, Houston, TX 77030, USA; XLiu10@mdanderson.org (X.-D.L.); EJonasch@mdanderson.org (E.J.); 9 Lineberger Comprehensive Cancer Center, University of North Carolina at Chapel Hill, Chapel Hill, NC 27514, USA; chrsmit@email.unc.edu (C.C.S.); benjamin_vincent@med.unc.edu (B.G.V.); 10 Department of Microbiology and Immunology, University of North Carolina at Chapel Hill, Chapel Hill, NC 27514, USA; 11 Navigate Biopharma Services, Inc., A Novartis Subsidiary, Carlsbad, CA 92008, USA; beiru.chen@navigatebp.com (B.C.); kristen.ruma@navigatebp.com (K.R.); nathan.roscoe@navigatebp.com (N.R.); jo.bender@navigatebp.com (J.B.); ju_young.kim@navigatebp.com (J.Y.K.); christine.vaupel@navigatebp.com (C.V.); jennifer.bordeaux@navigatebp.com (J.B.); 12 Thermo Fisher Scientific, Sacramento, CA 95605, USA; Joolz.Ward@ThermoFisher.com; 13 Rutgers Cancer Institute of New Jersey, New Brunswick, NJ 08901, USA; ganesash@cinj.rutgers.edu (S.G.); mayertm@cinj.rutgers.edu (T.M.M.); 14 Department of Medicine, Rutgers Robert Wood Johnson Medical School, Rutgers University, New Brunswick, NJ 08901, USA; 15 Department of Pathology, Rutgers Robert Wood Johnson Medical School, Rutgers University, New Brunswick, NJ 08901, USA; gr338@cinj.rutgers.edu; 16 Curriculum in Bioinformatics and Computational Biology, Computational Medicine Program, University of North Carolina at Chapel Hill, Chapel Hill, NC 27514, USA; 17 Nashville Veterans Affairs Medical Center, Nashville, TN 37212, USA

**Keywords:** renal cell carcinoma, PD-1, PD-L1, biomarkers, immune checkpoint inhibitors

## Abstract

**Simple Summary:**

Immune checkpoint inhibitor (ICI) therapy has proven effective for many cancer patients, but predicting which patients with renal cell carcinoma (RCC) will respond has been challenging. We analyzed clinical characteristics and molecular parameters of a cohort of patients with RCC treated with anti-programmed death 1 (PD-1)/PD-L1 therapy to determine factors that correlate with patient outcome. We found that the composition of circulating immune cells in the blood, development of immune-related toxicities, and gene expression patterns within the tumor correlate with patient response. In addition, we see that high expression of PD-L1 and lower numbers of unique T cell clones in RCC tumors are associated with improved survival. In summary, our findings corroborate previously published work and introduce new potential factors impacting response to ICI therapy that deserve further investigation.

**Abstract:**

Predicting response to ICI therapy among patients with renal cell carcinoma (RCC) has been uniquely challenging. We analyzed patient characteristics and clinical correlates from a retrospective single-site cohort of advanced RCC patients receiving anti-PD-1/PD-L1 monotherapy (N = 97), as well as molecular parameters in a subset of patients, including multiplexed immunofluorescence (mIF), whole exome sequencing (WES), T cell receptor (TCR) sequencing, and RNA sequencing (RNA-seq). Clinical factors such as the development of immune-related adverse events (odds ratio (OR) = 2.50, 95% confidence interval (CI) = 1.05–5.91) and immunological prognostic parameters, including a higher percentage of circulating lymphocytes (23.4% vs. 17.4%, *p* = 0.0015) and a lower percentage of circulating neutrophils (61.8% vs. 68.5%, *p* = 0.0045), correlated with response. Previously identified gene expression signatures representing pathways of angiogenesis, myeloid inflammation, T effector presence, and clear cell signatures also correlated with response. High PD-L1 expression (>10% cells) as well as low TCR diversity (≤644 clonotypes) were associated with improved progression-free survival (PFS). We corroborate previously published findings and provide preliminary evidence of T cell clonality impacting the outcome of RCC patients. To further biomarker development in RCC, future studies will benefit from integrated analysis of multiple molecular platforms and prospective validation.

## 1. Introduction

Over the past decade, immune checkpoint inhibitors (ICIs), including antibodies against the programmed death 1 (PD-1) receptor, its ligand (PD-L1), and cytotoxic T-lymphocyte-associated protein 4 (CTLA-4), have become a mainstay of treatment against cancer. Patients with metastatic renal cell carcinoma (RCC) have overall response rates (ORRs) to single-agent PD-1/PD-L1 blockade in the first- and second-line setting of approximately 16–34% [1,2,3,4]. The current standard of care in the frontline setting is combination therapy using anti-PD-1/PD-L1 with either anti-CTLA-4 or a vascular endothelial growth factor (VEGF)-targeting agent, which yields ORRs of 40–60% [4,5,6,7,8,9]. 

Predicting response to ICI therapy in patients with RCC has proven to be difficult. The predictive value of tumor PD-L1 expression and mutational burden (TMB), which are used as companion diagnostic biomarkers for other tumor types, remains equivocal in RCC, with a number of studies demonstrating no correlation with response [2,3,6,7,9,10,11]. Results of standard clinical tests on peripheral blood, including elevated absolute lymphocyte count (ALC) [12], or lower absolute neutrophil count (ANC) [12,13], neutrophil-to-lymphocyte ratio (NLR) [12,13,14,15], and monocyte-to-lymphocyte ratio (MLR) [14], have been associated with better response in solid tumors but not prospectively validated. In addition, the relationship between body mass index (BMI) and response is still disputable, with studies demonstrating improved response and survival in RCC patients with higher BMI [16,17] and others showing the opposite [18].

Molecular studies have shed light on the biological response behind anti-PD-1 monotherapy, such as the presence of endogenous retroviruses [19,20] and differential expression of gene signatures, including T cell effector function [10], interferon (IFN) or tumor necrosis factor (TNFα) signaling [21], and metabolic gene signatures [22]. In the randomized trials IMmotion150 and IMmotion151, molecular signatures of response were assessed in treatment-naïve patients who received sunitinib, the combination of atezolizumab (PD-L1 antibody) and bevacizumab (vascular endothelial growth factor (VEGF) antibody), or, in the case of IMmotion150, atezolizumab monotherapy [10,23]. A T effector signature correlated with response in both ICI monotherapy and combination therapy arms, while the angiogenic signature correlated with response in anti-VEGF monotherapy and combination therapy. Furthermore, responders to atezolizumab monotherapy generally had lower inflammatory myeloid signatures and higher T effector signatures, while patients with high suppressive myeloid signatures were more likely to achieve response if VEGF inhibition was combined with checkpoint inhibition [10]. More in-depth analysis from IMmotion150 further categorized RCC patients based on integration of various molecular parameters into seven molecular subsets, which appeared to have differential clinical outcomes to sunitinib versus atezolizumab plus bevacizumab [23].

Other investigations have attempted to correlate genetic drivers of RCC with response to ICI. The role of *PBRM1*, the second most commonly mutated gene in clear cell RCC (ccRCC) and a component of the chromatin remodeling complex, is heavily contested, with several studies yielding mixed results [11,24,25,26,27]. Recently, Braun et al. analyzed 592 ccRCC samples from patients in prospective trials of PD-1 blockade using whole exome sequencing (WES), RNA-sequencing (RNA-seq), and immunofluorescence (IF) analysis [11]. The authors found that although TMB and CD8+ T cell infiltration do not correlate with response, additional chromosomal abnormalities are specifically associated with response or resistance to anti-PD-1 monotherapy. For example, chromosomal loss of 9p21.3 was associated with decreased response among tumors with high CD8+ T cell infiltration. In addition, truncating mutations in *PBRM1* were associated with higher angiogenesis gene expression and lower IL6-JAK-STAT3 signaling, as well as improved survival with anti-PD-1 monotherapy [11,24,25].

In this study, we evaluated an institutional cohort of patients with RCC who received single-agent anti-PD-1/PD-L1 to study both clinical and molecular correlates of response. In our cohort of 94 patients, we analyzed clinical characteristics and laboratory data during the course of ICI treatment. Our study shows the feasibility of performing multiple molecular analyses, including mIF, WES, TCR sequencing, and RNA-seq, on a biomarker cohort created from available archived tumor samples. 

## 2. Materials and Methods

### 2.1. Patient Population and Data Collection

**Figure 1:**
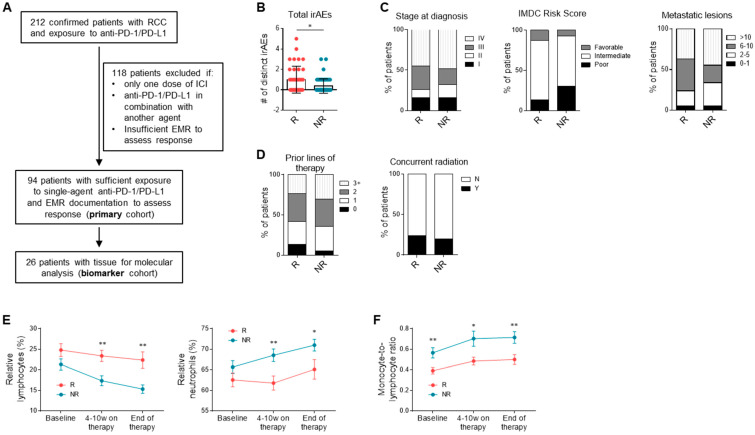
Clinical correlates and response to single-agent anti-PD-1/PD-L1. (**A**) Flow diagram depicting subdivision of primary and biomarker patient cohorts. (**B**) Number of immune-related adverse events (irAEs) experienced by responders and non-responders. Data shown are averages ± standard deviation (SD). * *p* < 0.05, unpaired Mann–Whitney U-test (*n* = 38, 56). (**C**) Stage at diagnosis, International Metastatic RCC Database Consortium (IMDC) risk score, and number of metastatic lesions of at initiation of ICI therapy. (**D**) Prior lines of therapy before ICI therapy, as well as concurrent radiation during ICI therapy. (**E**) Percentage of lymphocytes (two-way analysis of variance (ANOVA): response effect, *p* < 0.0001; time effect, *p* = 0.012; interaction, *p* = 0.44) and neutrophils (response effect, *p* = 0.0002; time effect, *p* = 0.063; interaction, *p* = 0.55) in peripheral blood of responders compared to non-responders at baseline, 4 to 10 weeks of therapy, and end of therapy (*n* = 37, 53). Data shown are averages ± SEM. * *p* < 0.05, ** *p* < 0.01, post-hoc two-tailed unpaired Welch’s *t* test, uncorrected for multiple comparisons. (**F**) Monocyte-to-lymphocyte ratio (MLR) (response effect, *p* < 0.0001; time effect, *p* = 0.047; interaction, *p* = 0.91) in responders compared to non-responders (*n* = 36, 52).

Patients with RCC who had been treated with single-agent anti-PD-1/PD-L1 at Vanderbilt University Medical Center (VUMC) between 2007 and 2017 were identified under an investigator review board (IRB)-approved protocol and verified to have sufficient documentation to assess response to therapy, defined by a minimum of a baseline and three-month computed tomography (CT) scan after initiating ICI therapy (Figure 1A). Objective response was evaluated by investigators. Of the 94 patients, 18 had available archived formalin-fixed, paraffin-embedded (FFPE) tumor tissue specimens at VUMC. These specimens and their matched normal samples, when available, combined with eight external tumor specimens from Rutgers University, were used for molecular studies in the biomarker cohort. A total of 26 patients were represented by 21 primary and 16 metastatic tumor samples.

### 2.2. DNA and RNA Extraction

DNA and RNA were extracted from FFPE RCC and normal tissue using the Maxwell 16 FFPE Plus LEV DNA Purification Kit (Promega (Madison, WI, USA) #AS1135) and RNA Purification Kit (Promega #AS1260) respectively, on the Promega Maxwell 16 instrument.

### 2.3. mIF Immunohistochemistry

#### 2.3.1. Immunofluorescence Staining

Antibody validation for the PD-1, PD-L1, and cytokeratin panel was performed as previously indicated in Johnson et al. [28] and can be found in Table S1. The CD4, CD8, CD25, FoxP3, Ki67, cytokeratin panel was performed using a fully automated staining protocol on the Bond Rx (Leica). Slides were dewaxed using the Bond Rx followed by antigen retrieval in Epitope Retrieval Solution 2 (Leica) buffer at 95 °C for 20 min. Primary antibodies were incubated for 1 h, detected with either EnVision+ HRP Mouse or EnVision+ HRP Rabbit for 30 min and slides were heat-cycled in ER1 (Leica) buffer for 20 min at 95 °C after each round of primary, secondary, and Opal fluorophore staining. Slides were stained with 1:100 dilution of mouse anti-CD4 (4B12, Agilent, Santa Clara, CA, USA), detected with Opal 520 (Akoya Biosciences, Marlborough, MA, USA), 1:400 dilution of mouse anti-CD8 (C8/144B, Agilent), detected with Opal 620 (Akoya Biosciences), 1:100 dilution of rabbit anti-FoxP3 (D2W8E, Cell Signaling Technology, Danvers, MA, USA), detected with Opal 540 (Akoya Biosciences), 1:400 dilution of rabbit anti-CD25 (SP176, Sigma Aldrich, St. Louis, MO, USA), detected with Opal 570 (Akoya Biosciences), 1:1000 dilution of mouse anti-Ki67 (MIB-1, Agilent), detected with Opal 650 (Akoya Biosciences), and 1:400 dilution of mouse anti-cytokeratin (AE1/AE3, Agilent), detected with Opal 690, and finally, incubated for 10 min with spectral 4′,6-diamidino-2-phenylindole (DAPI) (Akoya Biosciences).

#### 2.3.2. Sample Imaging

Fluorescence imaging was obtained as indicated in Johnson et al. [28]. The CD4/CD8/CD25/FoxP3/Ki67/CK assay was imaged using Vectra 3 software (Akoya Biosciences), where the whole slide was scanned at 4× for DAPI, fluorescein isothiocyanate (FITC), CY3, Texas Red, and Cy5, and an automated algorithm was used to enrich for areas with CD25 and FoxP3 staining. The images were then reviewed by a pathologist and adjusted to ensure tumor areas were included in the imaging before 20× multispectral images were acquired of up to 40 fields of view. Accepted images were processed with AQUA import tool (Navigate) to generate spectrally unmixed images for analysis.

### 2.4. WES and Analysis

Raw sequencing data, paired fastq files, were processed using Genome Analysis Toolkit (GATK) [29]. Briefly, reads were aligned using Burrows-Wheeler Aligner Maximum Exact Match (BWA-MEM) [30] against the hg38 human reference and duplicate reads were marked. Joint variant calling was performed using Haplotype Caller [31]. Analysis was performed using Terra [32] on Google Cloud Platform. Variants were annotated using Annotate Variation (ANNOVAR) [33] and filtered using standard filtering criteria to obtain high quality, likely somatic variants, and variant classification. The following filters were used: (1) quality filters: quality by depth, read position bias, (2) exclusion of non-exonic variants, (3) exclusion of variants with missing genotypes found in >25% of the samples, (4) exclusion of variants found in matched normal samples, (5) exclusion of synonymous variants or variants of unknown function, and (6) max population frequency < 0.001 using the following population datasets: ExAC [34], gnomAD [35], 1000 G [36], and ESP6500 [37].

Driver gene-filtering was performed as follows. Genes implicated in prior studies [38] and implicated in OncoKB [39] were retained. For the remaining genes, we applied the following filters for downstream analysis: (1) non-synonymous/synonymous ratio > 2.5 or frequency of frameshifts >33%, (2) transcript size <15,000 amino acids, and (3) variability ≤0.06 (number of variants as a function of variant size in the ESP6500 database [37]). For the mutation heatmap, genes were sorted based on frequency, and variants were color-coded based on their effect.

### 2.5. TCR Sequencing and Analysis

Analysis of TCR sequencing data was performed using MIGEC and VDJtools [40]. Briefly, unique molecular barcodes (UMIs) were used to create consensus reads associated with each molecule, reducing polymerase chain reaction (PCR) and sequencing errors. Hierarchical clustering was performed on TCR genes to verify that samples from the same patient clustered together and to detect cross-contamination. TCR diversity was correlated with clinical variables, and median diversity was used as a cutoff for PFS curves.

### 2.6. RNA-seq and Analysis

RNA-seq libraries were generated with the Illumina TruSeq RNA Access kit, according to the manufacturer’s protocol. Sequencing was performed using 2 × 100 read chemistry on an Illumina HiSeq 2500 system. Alignment of FASTQ files was performed using STAR v2.4.2a with default arguments, with downstream quantification performed using Salmon v0.8.2 in quantification mode. Quality control of BAM files was performed using Picard Tools v1.86 “CollectRnaSeqMetrics”. Gene count matrices were analyzed using DESeq2 [41], and normalized gene expression counts were obtained. We performed principal component analysis (PCA) to identify groupings or outliers from the data and compared them to clinical covariates. Differential analysis was performed to identify differential genes between responders and non-responders (adjusted *p*-value (Benjamini–Hochberg) < 0.1). Angiogenic, T effector, inflammatory myeloid signatures, and the expanded immune and antigen-presenting gene panel were defined as previously published [10]. The contribution of immune cell types in each of the samples was identified using deconvolution methods [42]. Clear cell subtypes ccA and ccB were defined by the ClearCode34 gene [43].

### 2.7. Statistical Analysis

Analyses of clinical characteristics were performed using GraphPad Prism v6. Comparisons of clinical characteristics between responders and non-responders were carried out using two-way analysis of variance (ANOVA) with post-hoc unpaired Welch’s *t* test at individual time points for laboratory data, Mann–Whitney U-tests for continuous and ordinal variables with non-normal distributions (i.e., number of irAEs), and Pearson χ2 or Fisher’s exact tests for categorical variables (i.e., previous nephrectomy). Survival analysis was performed using Kaplan–Meier estimation of survival functions followed by log-rank testing. All tests were two-sided, and a *p*-value less than 0.05 was statistically significant. Statistical analysis did not correct for multiple comparisons.

Analyses using molecular platforms and clinical variables were performed using R v3.6.1 (https://www.r-project.org/, accessed on 13 May 2020) and GraphPad Prism v6 (San Diego, CA, USA). For mIF, only primary clear cell RCC samples were included in comparative analyses to prevent confounding due to tissue or tumor type, and comparison of mIF data was evaluated by Mann–Whitney U-tests. Gene-level mutations obtained from WES and gene expression data were used to identify associations with clinical covariates. Survival analysis was used to identify associations with PFS, and differential analysis was used to identify associations with clinical response.

## 3. Results

### 3.1. Study Population

Of the 212 patients who were verified to have RCC and treated with any ICI therapy, 94 patients had sufficient electronic medical record (EMR) documentation and treatment with single-agent anti-PD-1 or PD-L1 therapy to assess response (Figure 1A). The demographic and treatment information for these patients are summarized in Table 1 (Table S6). Clinical characteristics were similar to those reported in trials investigating single-agent anti-PD-1/PD-L1, though our cohort had a considerably lower proportion of patients in the favorable International Metastatic RCC Database Consortium (IMDC) risk group [2,15]. Thirty-eight patients (40.4%) were considered as responders (complete response (CR), partial response (PR), mixed response), and 56 (59.6%) as non-responders (stable disease (SD) and progressive disease (PD)). Median PFS and overall survival (OS) of all patients was 6.6 months (95% confidence interval (CI): 4.4–8.7) and 23.5 months (20.4–34.1), respectively. The clinical characteristics for the biomarker cohort are detailed in Table S2 and were enriched for responders (50%) compared to the primary cohort. 

### 3.2. Clinical Correlates and Response to Anti-PD-1/PD-L1

Consistent with previous reports, the number of irAEs experienced while on ICI therapy was higher in responders than non-responders (*n* = 38, 56; *p* = 0.012) (Figure 1B), and the odds of response were increased in patients who experienced at least one irAE compared to those who experienced none (odds ratio (OR) = 2.50, 95% CI = 1.057–5.911) (Table S3). Among patients who experienced an irAE, the type of irAE that occurred, the highest grade of irAE experienced, and the percentage of patients requiring oral and/or intravenous steroid administration did not differ significantly between responders and non-responders (Table S3). Stage at diagnosis, IMDC risk score, number of metastatic lesions at initiation of ICI therapy, number of previous lines of systemic therapy, and concurrent radiation with ICI therapy were not associated with the probability of objective response (Figure 1C, D). Although the number of metastatic sites did not differ between responders and non-responders, the presence of pancreatic metastasis correlated with decreased likelihood of response (OR = 0.257, 95% CI = 0.0683–0.968) (Table S4). Other demographic (age, gender, BMI), and treatment characteristics (previous nephrectomy, radiation, antiangiogenic agent, mammalian target of rapamycin (mTOR) inhibitor, or IL-2 therapy) were not significantly different between groups (Figure S1A,B).

Evaluation of patient laboratory values showed that while no difference in percentage of lymphocytes (*n* = 37, 53; 24.8% vs. 21.3%, *p* = 0.10) or neutrophils (*n* = 37, 53; 62.5% vs. 65.7%, *p* = 0.18) of total leukocytes was seen at time of ICI initiation, there was a significantly higher percentage of lymphocytes (23.4% vs. 17.4, *p* = 0.0015) and lower percentage of neutrophils (61.8% vs. 68.5%, *p* = 0.0045) in responders compared to non-responders early during the course of ICI therapy that was sustained until the end of therapy (*p* = 0.0030, *p* = 0.038) (Figure 1E). Additionally, MLR was lower in responders compared to non-responders throughout all measured timepoints (*n* = 36, 52; two-way ANOVA; response effect *p* < 0.0001, time effect *p* = 0.047, interaction *p* = 0.91) (Figure 1F).

### 3.3. PD-L1 Expression and Immune Milieu

**Figure 2:**
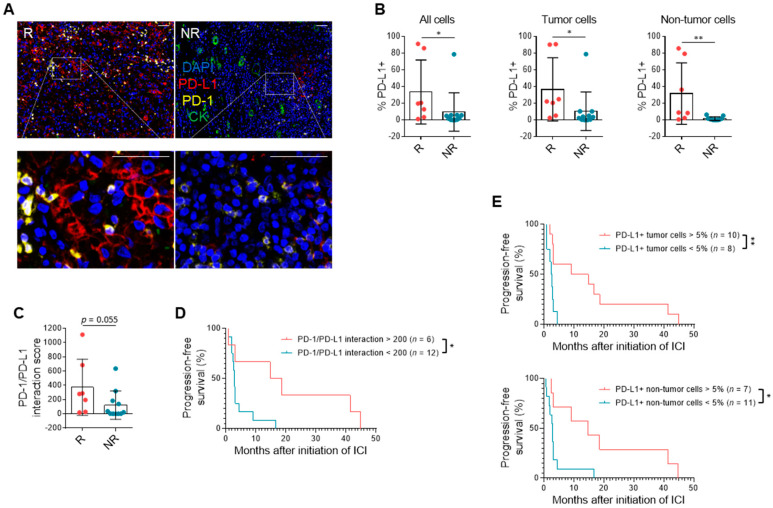
PD-L1 expression is associated with response to anti-PD-1/PD-L1 therapy. (**A**) Representative mIF images of primary tumors stained for DAPI (blue), PD-L1 (red), PD-1 (yellow), and cytokeratin (CK) (green) from a responder and non-responder. Scale bar: 50 μm. (**B**) Quantification of PD-L1 expression on all cells, tumor cells, and non-tumor cells in primary tumors from responders compared to non-responders. Data shown are averages ± SD. * *p* < 0.05, ** *p* < 0.01, unpaired Mann–Whitney U-test (*n* = 7, 10). (**C**) PD-1/PD-L1 interaction scores in responders versus non-responders. Data shown are averages ± SD. Unpaired Mann–Whitney U-test (*n* = 7, 10). (**D**) PFS of patients based on PD-1/PD-L1 interaction score threshold of 200. * *p* < 0.05, log-rank test (*n* = 6, 12). (**E**) PFS of patients based on PD-L1 expression of tumor (*n* = 10, 8) or non-tumor cells (*n* = 7, 11) with a threshold of 5%. * *p* < 0.05, ** *p* < 0.01, log-rank test.

To further our understanding of the biology underlying response to PD-1/PD-L1 blockade in patients with RCC, we performed multiplatform molecular profiling in the biomarker cohort (Table S7). PD-L1 expression was assessed on tumor, non-tumor, and all cells. The percentage of PD-L1-expressing cells was increased in responders compared to non-responders, most significantly among non-tumor cells (*n* = 7, 11; *p* = 0.0058) (Figure 2A,B). Prior work in melanoma suggests that the density of both PD-1 and PD-L1 expression, quantified by the interaction score, is a stronger predictor than PD-L1 alone [28]. Although PD-1 expression in non-tumor cells was not different between responders and non-responders (Figure S2A), the PD-1/PD-L1 interaction score was non-significantly higher in responders (*n* = 7, 11; *p* = 0.055) (Figure 2C) and a score over 200 correlated with improved PFS (*n* = 6, 12; hazard ratio (HR) = 0.38, 95% CI = 0.11–0.70) (Figure 2D). Regardless of cell type measured, PD-L1 expression correlated with PFS when using a threshold of >5% when measured on either tumor cells (*n* = 10, 8; HR = 0.31, 95% CI = 0.047–0.48) or non-tumor cells (*n* = 7, 11; HR = 0.36, 95% CI = 0.098–0.67) (Figure 2E). When looking across all cells, higher overall PD-L1 expression correlated with improved survival, which was significant when using a cutoff of 10% of all cells (*n* = 6, 12; HR = 0.30, 95% CI = 0.079–0.53) (Figure S2B).

To better understand the presence and impact of other cells in the tumor immune microenvironment in RCC and response to immunotherapy, mIF measured both immune stimulatory and suppressive components. The amount of total, CD4+, and CD8+ T cell infiltration did not correlate with response (Figure S2C). Immune-suppressive components in the RCC microenvironment such as T regulatory cells (Tregs), macrophages, myeloid-derived suppressor cells (MDSCs), and cells expressing indoleamine 2,3-dioxygenase (IDO1) were lower in responders, though not significantly (*n* = 7, 10; *p* = 0.16, 0.29, 0.39, 0.41) (Figure S2D).

### 3.4. TMB and Driver Mutations Do Not Correlate with ICI Response

**Figure 3:**
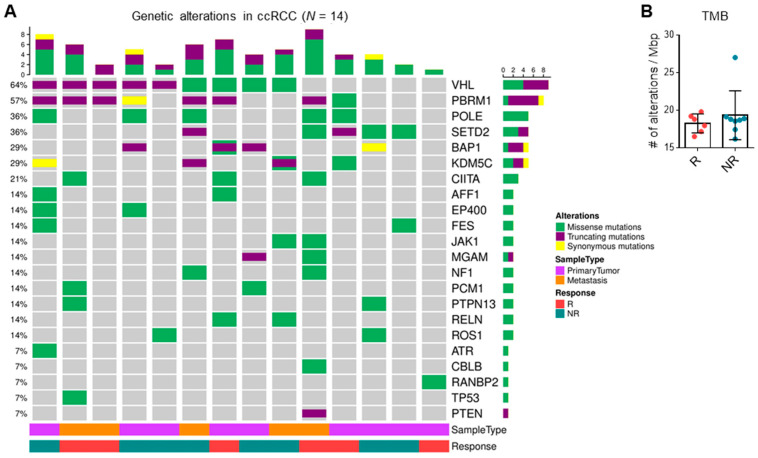
Tumor mutational burden (TMB) and driver mutations do not correlate with response. (**A**) Quantification of missense, truncating, and synonymous mutations in top 22 altered genes found in clear cell RCC samples. (**B**) TMB of responders (*n* = 6) compared to non-responders (*n* = 8) calculated based on all unfiltered variants. Data shown are averages ± SD.

WES was performed in patients who had matched primary tumor, metastasis, and/or adjacent normal tissue. Matched samples from the same patient showed a high degree of similarity quantified by the Jaccard index and clustered together as expected (Table S8). Consistent with previous reports, the classical driver mutations associated with ccRCC, including alterations in VHL, PBRM1, BAP1, and SETD2, were identified in tumor samples. Truncating mutations were the most common, followed by missense and synonymous mutations (Figure 3A). However, responders and non-responders did not cluster based on WES analysis, and no single gene or mutation significantly correlated with response. Non-synonymous PBRM1 mutations trended towards response (OR = 15.00) but were not statistically significant (*p* = 0.10). Additionally, TMB calculated based on all unfiltered variants did not correlate with response to therapy (Figure 3B). Thus, tumor mutational profile did not predict likelihood of response to ICI.

### 3.5. TCR Clonal Diversity Does Not Correlate with Response but May Impact Survival

**Figure 4:**
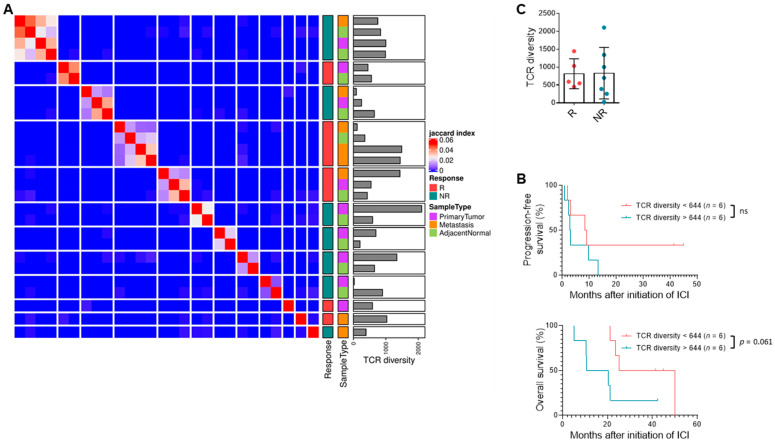
TCR clonal diversity does not correlate with response but may impact survival. (**A**) Heatmap depicting unsupervised clustering analysis of Jaccard index representing similarity among primary tumor, metastatic tumor, and adjacent normal samples from 12 patients in biomarker cohort based on TCR sequencing. (**B**) PFS and OS of patients with low versus high intra-tumoral TCR diversity based on median cutoff of 644 clonotypes. Log-rank test (*n* = 6, 6). (**C**) Quantification of TCR diversity in primary tumors from responders compared to non-responders. Data shown are averages ± SD.

Lower TCR diversity may represent purposeful expansion of specific tumor antigen-driven TCR clones, indicating an existing anti-tumor T cell response that can be further enhanced with immunotherapy. Previous studies in ccRCC have demonstrated that a polyclonal infiltrating T cell population is indicative of an exhausted, poorly cytotoxic T cell phenotype compared to an oligoclonal population [44]. As expected, unsupervised clustering of TCR clones in our samples showed that TCRs were not generally shared across patients, and multiple samples from a single individual clustered together (Figure 4A, Table S9). Although TCR diversity was not significantly different in objective response (*n* = 5, 7) (Figure 4B), a lower TCR diversity (<644 clonotypes) among tumor-infiltrating lymphocytes suggested improved survival (*n* = 6, 6; PFS HR = 0.49, 95% CI = 0.13–1.6; OS HR = 0.32, 95% CI = 0.055–0.98) (Figure 4C).

### 3.6. Gene Expression Patterns in RCC Suggest Response to Single-Agent Immunotherapy

**Figure 5:**
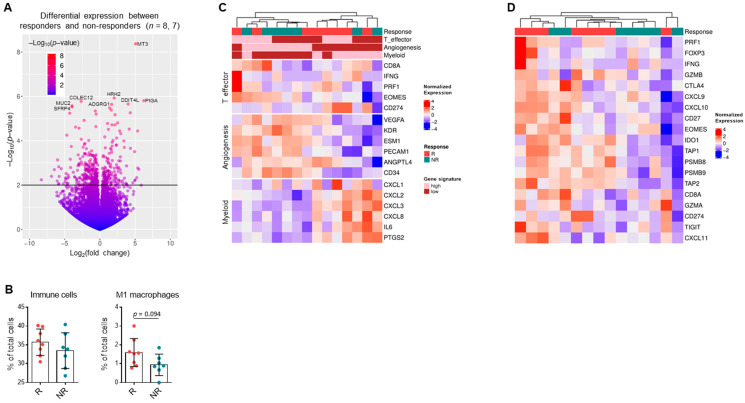
Gene expression patterns in RCC suggest response to single-agent immunotherapy. (**A**) Volcano plot depicting statistical significance against fold-change expression of differentially expressed genes from responders (*n* = 8) and non-responders (*n* = 7) in biomarker cohort based on RNA-seq data. Top eight differentially expressed genes labeled. (**B**) Percentages of all immune cells and M1 macrophages in bulk tumor samples from responders compared to non-responders based on deconvolution analysis. Data shown are averages ± SD, unpaired Mann–Whitney U-test (*n* = 8, 7). (**C**) Heatmap depicting clustering of tumor samples by T effector, angiogenesis, and myeloid signature scores. (**D**) Heatmap depicting clustering of tumor samples by expanded immune and antigen-presenting gene expression panel.

Prior work has suggested that gene expression signatures correlate with response to anti-PD-1/PD-L1 therapy [10,21,22]. Differential gene expression analysis in our study demonstrated genes that were significantly differentially expressed between responders and non-responders (*n* = 8, 7) (Figure 5A, Table S10) and gene set variation analysis revealed differentially expressed pathways, including immune and metabolic pathways, between responders and non-responders (Table S5). Furthermore, deconvolution analysis revealed a non-significantly higher proportion of tumor-infiltrating immune cells (*p* = 0.34), including M1 macrophages (*p* = 0.094), among responders compared to non-responders (Figure 5B). Consistent with mIF results, proportions of other immune cell types, including CD8+ T cells, were not different between responders and non-responders (Figure S3A). Evaluation using the previously published ClearCode34 gene set [43] showed that responders to PD-1/PD-L1 blockade tended to cluster together with a ccB profile, while non-responders were largely grouped under the ccA profile (Figure S3B).

Unsupervised clustering analysis showed that responders and non-responders tended to cluster separately based on key gene expression pathways, including angiogenesis, myeloid, and T effector signature scores previously defined by McDermott et al. [10] and shown in Figure 5C. Responders in this cohort tended to have a lower angiogenic and higher T effector signature. Expression of the expanded immune and antigen-presenting gene panel [10], which includes other stimulatory cytokines and immune checkpoint proteins in addition to the T effector signature, was upregulated in responders compared to non-responders (Figure 5D). In summary, previously identified gene expression signatures including angiogenic, T effector, expanded immune, and clear cell subtype (ClearCode34) gene signatures correlated with response to anti-PD-1/PD-L1 monotherapy.

## 4. Discussion

The results of this retrospective study identify clinical and laboratory characteristics associated with response to ICI in patients with RCC and explore how these characteristics relate to novel biomarker platforms. Patients who experienced at least one irAE were more likely to respond to ICI. These results replicate prior associations between irAE and patient response to ICI [16,17] and suggest that immune reactivity occurs not only at the site of the tumor but also at non-tumor tissue sites. While such association requires large-scale validation, irAE in patients with mixed response on imaging or concerns for pseudoprogression may aid in decision-making for clinicians. Patients with pancreatic metastases were less likely to respond to anti-PD-1/PD-L1. This is consistent with results reported by Singla et al., who have shown that pancreatic metastases of RCC are typically VEGF-driven and refractory to ICI treatment [45]. 

Patients with response to ICI commonly had higher lymphocyte and lower neutrophil percentages, corroborating work that has shown NLR at baseline and after several weeks on treatment as a predictor of response in RCC. Patients with higher NLR have lower ORR, PFS, and OS on ICI therapy [12]. These findings suggest that higher levels of suppressive myeloid cells induced by the tumor and lower levels of activated circulating lymphocytes contribute to the correlation between high NLR and poor response to ICI therapy. The clinical application of NLR awaits further prospective study.

PD-L1 testing has been fraught with challenges, including tumor heterogeneity, differing antibodies, varying percent expression cutoff, and differing target cell populations of the analysis. PD-L1 in this mIF assay showed correlation with response across cell types assessed, but, similar to prior studies [2,3,6,7,10], not all patients with response had expression of PD-L1 at the protein level. Unlike our findings, prior studies generally did not find an association between overall PD-L1 expression and response to ICI therapy. Potential reasons for these discrepant results include the fact that the PD-L1 antibody in our assay does not share the same clonality or manufacturer as those used in clinical trials and our staining and detection methods differ from traditional immunohistochemistry (IHC). The novelty of this study was the ability to look not only at PD-L1 but at other cells in the tumor immune microenvironment. In non-responders, there were higher immune suppressive components such as Tregs and macrophages, while in responders, higher total T cell infiltration was found. These data suggest that, in addition to PD-L1, the presence and function of cells in the microenvironment are associated with response to ICI. Larger validation studies incorporating additional cell types covering both immune effector as well as suppressive functions are likely to add to the understanding of response to ICI in RCC. 

Braun et al. recently used WES, RNA-seq, IF, and copy number analysis to study response in a similar clinical context as this study for patients with ccRCC receiving ICI therapy [11]. They utilized clinical correlates from patients enrolled on Checkmate010 and Checkmate025, with the majority of this group receiving nivolumab following VEGF-targeted therapy. Our biomarker cohort differs in that the majority of patients received anti-PD-1/PD-L1 monotherapy in the third or later line of treatment and that responses were durable in the responders (median PFS, 13.4 months) compared to non-responders (median PFS, 2.76 months). Similar to Braun et al., we found that TMB and CD8+ T cell infiltration were not associated with clinical response. We did not observe a significant correlation between PBRM1 mutations and response as Braun et al. did, and this may in part be due to differences in cohorts and inclusion of non-truncating PBRM1 mutations in correlative analyses. Additionally, Braun et al. performed copy number variation analysis that we did not, which showed that additional chromosomal aberrations can help discriminate response. 

Based on data from studies using ClearCode34 [43] and IMmotion150 [10], we reviewed gene expression patterns to determine if specific pathways or previously established signatures could be differentially enriched in this study. In our small cohort, patients with response demonstrated differentially expressed genes involved in inflammatory signaling and metabolic pathways. Response tended to correlate more with the ccB subtype, as well as lower angiogenic, higher T effector, and higher expanded immune gene signatures. This is the first study to date suggesting an association between clear cell subtype and response to ICI therapy. Previous studies utilizing patient cohorts not treated with checkpoint inhibitors demonstrated that ccB tumors tend to have a worse prognosis compared to ccA tumors [43]. Thus, our work suggests that ICI therapy may provide greater clinical benefit, specifically in patients with ccB tumors.

Compared to IMotion150, we observed similar patterns in responders with high T effector signature and high expanded immune infiltrate signature. In IMotion150, treatment-naïve patients with high T effector combined with high myeloid-suppressive signatures tended not to do as well with single-agent PD-L1 blockade [10]. In this cohort, low angiogenesis and high T effector signatures were more likely to respond, consistent with previously published studies [10,23]. While results between this study and IMotion150 were discrepant regarding ability to respond with a high myeloid signature, this may be due to differences in patient population and line of therapy. While the patients in IMotion150 were treatment-naïve, patients in this study had several lines of therapy between sample collection and ICI treatment. Standard of care treatments in RCC such as VEGF inhibition have been shown to alter the tumor immune microenvironment and enhance response to anti-PD-1/PD-L1 monotherapy [46]. Samples were from archival FFPE tissue, including prior nephrectomy samples when the biology of a patient’s response may have been very different. Sequential biopsy while on therapy and at time of progression would improve our understanding of the changes in the tumor microenvironment and immune milieu that we hypothesize may occur after treatment with VEGF inhibition or other therapies.

New data from this study are preliminary results suggesting that the presence of lower TCR clonality should be investigated further for its potential to distinguish patient outcome. This analysis suggests that having a lower TCR diversity may correlate with improved PFS and OS and specific tumor clonotypes may drive the response to anti-PD-1/PD-L1 monotherapy. Future directions include identifying the tumor antigens associated with these clonotypes. In our cohort, we did not find overall TMB and alterations in commonly mutated genes like PBRM1 to be associated with response. Thus, neoantigens derived from these common mutations may not be the primary drivers of the adaptive T cell response in RCC; instead, the role of other factors such as endogenous retroviral genes [19,20] or larger chromosomal abnormalities [11] should be studied in shaping the TCR repertoire.

We note that this study has limitations, including being conducted at a single site in a retrospective manner. The use of archival FFPE specimens may skew analyses due to suboptimal molecular quality, though many others have similarly utilized this preservation method for their studies. RCC is also characterized by intratumoral heterogeneity and thus a single tissue sample analyses likely underestimated the complex biology of response. Similarly, future studies would benefit from focused analyses of varying histologies including non-ccRCC such as papillary. Although we demonstrate patterns and further our understanding of the biology that correlate with response to ICI in RCC, we are unable to definitively classify responders and non-responders based on these data. The clinical and biomarker cohorts were both limited by number of patients and samples included in each. In the future, more rigorous analyses will benefit from larger cohorts and accounting for multiplicity of testing. While limited, ultimately, this study independently confirms gene expression data from previously published work while also showing the feasibility to study more than one biomarker in order to improve understanding behind the complex biology underlying response to ICI treatment in RCC.

## 5. Conclusions

Response to ICI therapy remains challenging to predict in RCC, but these data build upon previous work and suggest that PD-L1 staining alone does not give sufficient information to predict response [11,24]. Checkpoint inhibitors elicit a complex biology that will require a combination of biomarkers to predict response. Platforms analyzing TCR diversity, gene expression, multiplex IHC or IF, and chromosomal alterations or endogenous retroviruses will need to be assessed in large prospective clinical trials moving forward with the goals of developing sensitive and specific biomarkers that can be used in the clinic.

## Figures and Tables

**Table 1:** Clinical characteristics of primary cohort of patients with RCC.

Clinical Characteristic	Primary Cohort, *n* = 94	Responders (CR, PR, Mixed), *n* = 38	Non-Responders (PD, SD), *n* = 56
Best response to ICI therapy (%)			
CR	2 (2.1)	2 (5.3)	0 (0.0)
PR	23 (24.5)	23 (60.5)	0 (0.0)
SD	18 (19.1)	0 (0.0)	18 (32.1)
PD	38 (40.4)	0 (0.0)	38 (67.9)
Mixed	13 (13.8)	13 (34.2)	0 (0.0)
Median age at initiation of ICI (range), year	63 (27–82)	62 (27–79)	63 (31–82)
Sex (%)			
Male	71 (75.5)	30 (78.9)	41 (73.2)
Female	23 (24.5)	8 (21.1)	15 (26.8)
Stage at diagnosis (%)			
I	15 (16.0)	6 (15.8)	9 (16.1)
II	13 (13.8)	4 (10.5)	9 (16.1)
III	22 (23.4)	11 (28.9)	11 (19.6)
IV	44 (46.8)	17 (44.7)	27 (48.2)
Histology			
Clear cell	79 (84.0)	32 (84.2)	47 (83.9)
Papillary	4 (4.3)	1 (2.6)	3 (5.4)
Sarcomatoid	2 (2.1)	1 (2.6)	1 (1.8)
Chromophobe	2 (2.1)	0 (0.0)	2 (3.6)
Undifferentiated	7 (7.4)	4 (10.5)	3 (5.4)
IMDC risk group (%)			
Favorable	9 (9.6)	5 (13.2)	4 (7.1)
Intermediate	63 (67.0)	28 (73.7)	35 (62.5)
Poor	22 (23.4)	5 (13.2)	17 (30.4)
Previous therapies (%)			
Nephrectomy	90 (95.7)	35 (92.1)	55 (98.2)
Radiation	32 (34.0)	13 (34.2)	19 (33.9)
Anti-angiogenic agent	81 (86.2)	30 (78.9)	51 (91.1)
mTOR inhibitor	25 (26.6)	10 (26.3)	15 (26.8)
High-dose IL-2	22 (23.4)	11 (28.9)	11 (19.6)
ICI agent (%)			
Nivolumab	79 (84.0)	28 (73.7)	51 (91.1)
Atezolizumab	15 (16.0)	10 (26.3)	5 (8.9)
ICI line of therapy (%)			
First-line	8 (8.5)	5 (13.2)	3 (5.4)
Second-line	28 (29.8)	11 (28.9)	17 (30.4)
Third-line	32 (34.0)	13 (34.2)	19 (33.9)
Fourth-line+	26 (27.7)	9 (23.7)	17 (30.4)
Median duration of ICI therapy (range), days	189 (12–1637)	329 (28–1637) ****	98 (12–769) ****
Median survival (95% CI), months			
PFS	6.6 (4.4–8.7)	11.1 (9.0–23.6) ^####^	3.1 (2.7–5.7) ^####^
OS	23.5 (20.4–34.1)	43.6 (29.4–not reached) ^####^	16.4 (10.6–23.0) ^####^

**** *p* < 0.0001, two-tailed Mann-Whitney U test. #### *p* < 0.0001, log-rank test, Abbreviations: CR, complete response; PR, partial response; SD, stable disease; PD, progression of disease; ICI, immune checkpoint inhibitor; IMDC, International Metastatic RCC Database Consortium; irAE, immune-related adverse event; CI, confidence interval; PFS, progression-free survival; OS, overall survival.

## Data Availability

The data presented in this study are available in the Supplementary Materials.

## Supplementary Materials

The following are available online at https://www.mdpi.com/2072-6694/13/6/1475/s1, Figure S1: Patient characteristics and prior therapies, Figure S2: PD-L1 expression is associated with survival, Figure S3: Gene expression patterns in RCC suggest response to single-agent immunotherapy, Table S1: Antibody panels used in multiplexed immunofluorescence assay, Table S2: Clinical characteristics of biomarker cohort of patients with RCC, Table S3: irAEs during ICI therapy in primary patient cohort, Table S4: Sites of metastasis prior to initiation of ICI in primary patient cohort, Table S5: Selected differentially expressed pathways from gene set variation analysis. Table S6: Clinical laboratory data from primary cohort, Table S7: mIF data from biomarker cohort, Table S8: Filtered variant data from biomarker cohort, Table S9: TCR-sequencing data from biomarker cohort, Table S10: Normalized gene expression counts from biomarker cohort.
