# Supplementary material for: Clinical Features and Multiplatform Molecular Analysis Assist in Understanding Patient Response to Anti-PD-1/PD-L1 in Renal Cell Carcinoma
Source: Cancers (Basel). 2021 Mar 23;13(6):1475. doi: 10.3390/cancers13061475 (PMC8004696; doi:10.3390/cancers13061475)
Supplement: Supplementary file 1 [file cancers-13-01475-s001.zip › 031421 Supplemental Figures and Tables S1-5.pdf]

Figure S1

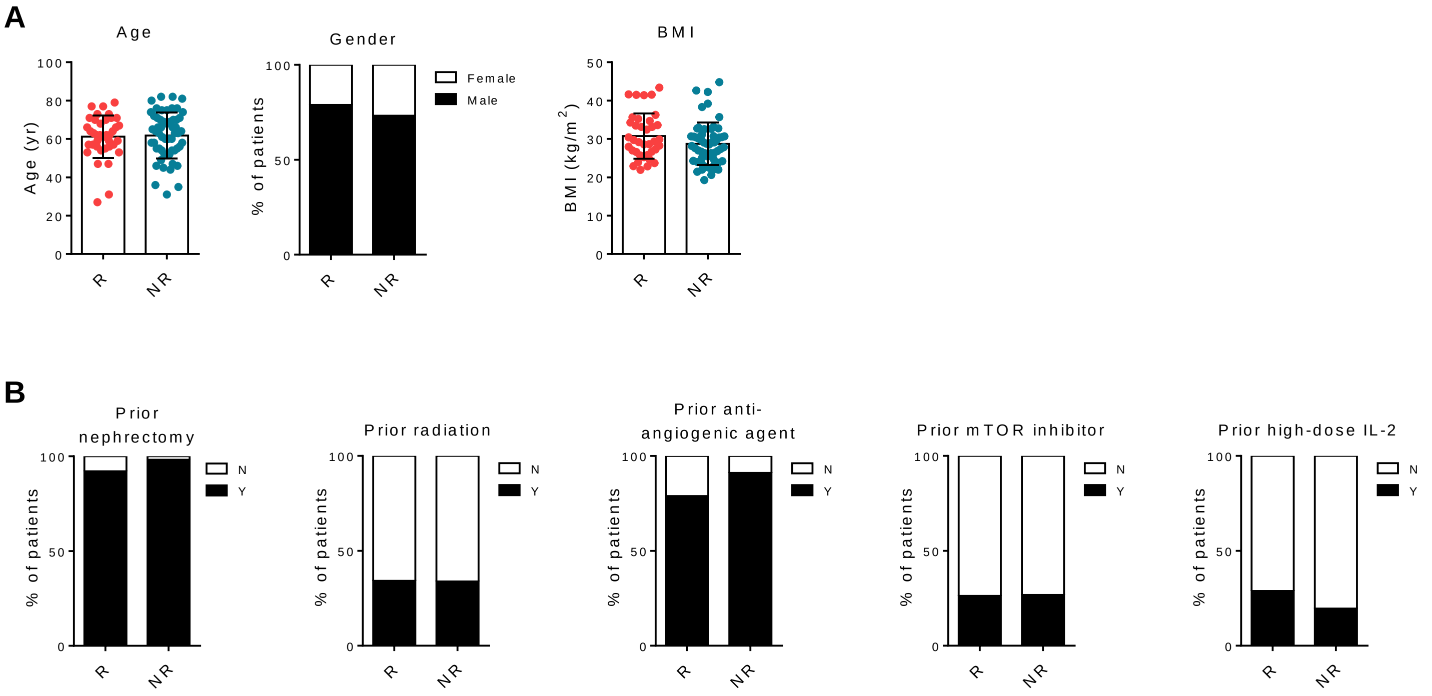
A
Age
Age (yr)
Gender
% of patients
Female
Male
BMI
BMI (kg/m2)
R
NR
B
Prior nephrectomy
Prior radiation
Prior anti-angiogenic agent
Prior mTOR inhibitor
Prior high-dose IL-2
N
Y

Figure S2

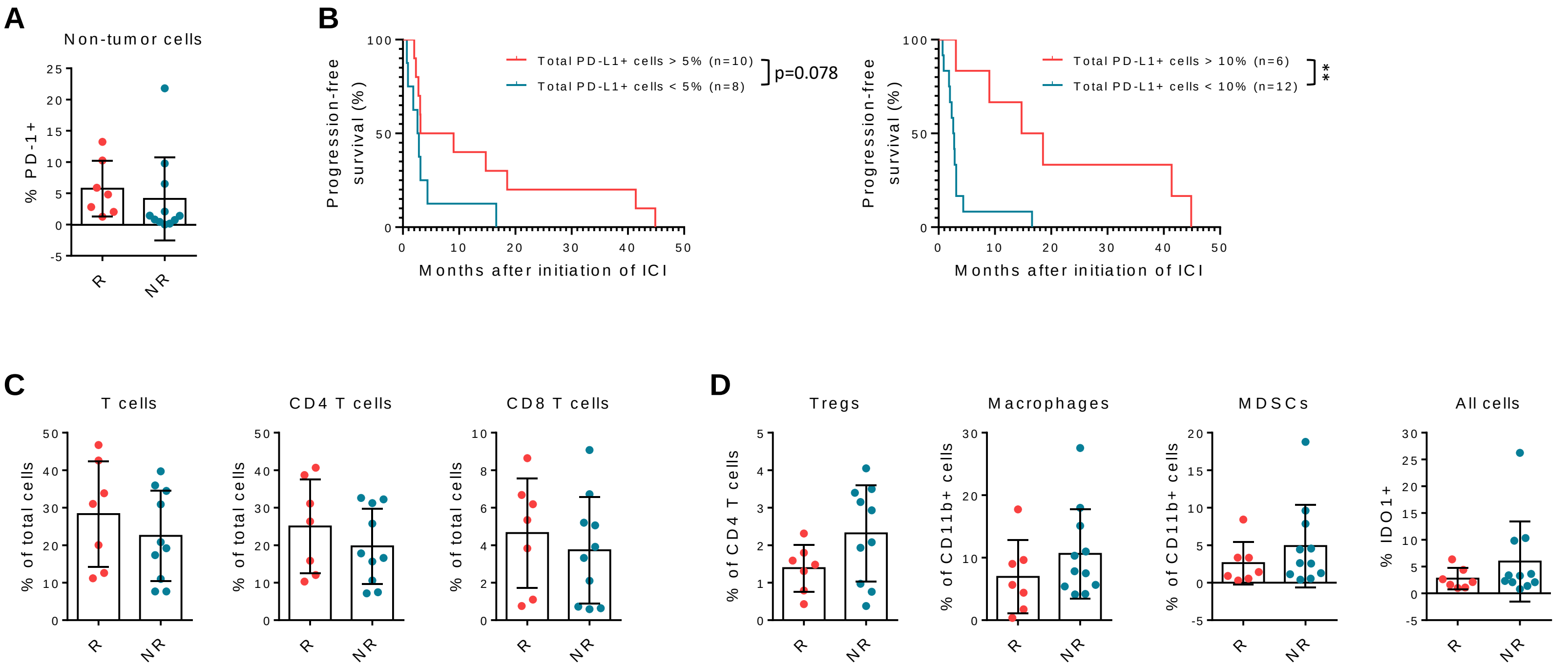
A
Non-tumor cells
% PD-1+
R
NR
B
Progression-free survival (%)
Months after initiation of ICI
Total PD-L1+ cells > 5% (n=10)
Total PD-L1+ cells < 5% (n=8)
p=0.078
Total PD-L1+ cells > 10% (n=6)
Total PD-L1+ cells < 10% (n=12)
**
C
T cells
CD4 T cells
CD8 T cells
% of total cells
D
Tregs
Macrophages
MDSCs
All cells
% of CD4 T cells
% of CD11b+ cells
% IDO1+

Figure S3

A

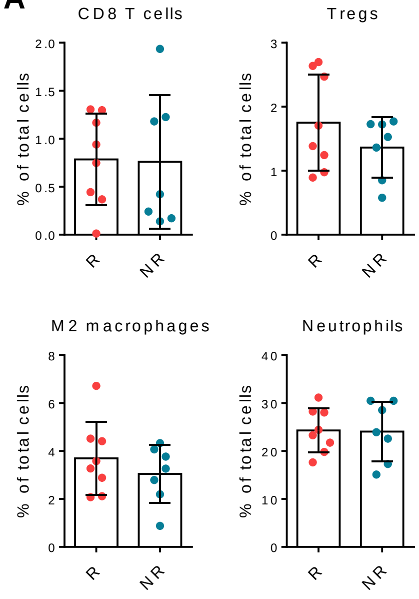
CD8 T cells
Tregs
M2 macrophages
Neutrophils
% of total cells
R
NR

B

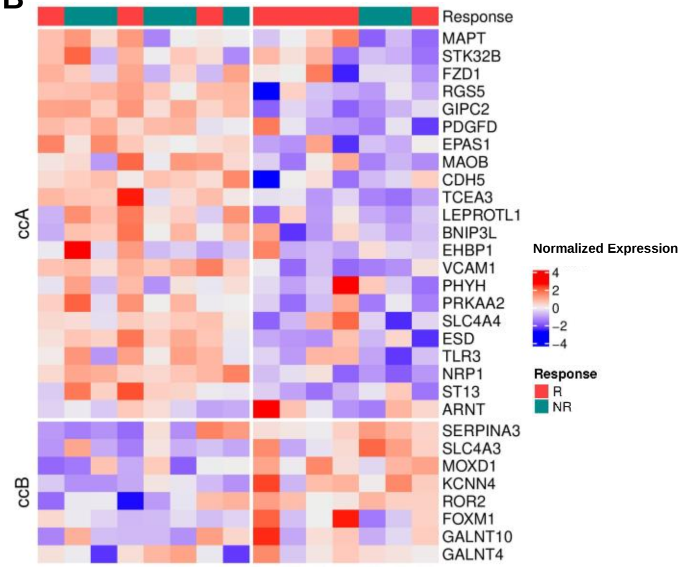
Response
MAPT
STK32B
FZD1
RGS5
GIPC2
PDGFD
EPAS1
MAOB
CDH5
TCEA3
LEPROTL1
BNIP3L
EHBP1
VCAM1
PHYH
PRKAA2
SLC4A4
ESD
TLR3
NRP1
ST13
ARNT
SERPINA3
SLC4A3
MOXD1
KCNN4
ROR2
FOXM1
GALNT10
GALNT4
ccA
ccB
Normalized Expression
Response
R
NR

Table S1. Antibody panels used in multiplexed immunofluorescence assay

Panel	Antibody	Clone	Secondary	Fluorophore
PD-1/PD-L1/CK	PD-1	NAT105	Mouse EnVision+ HRP	TSA+Cy3.5
	PD-L1	E1L3N	Rabbit EnVision+ HRP	TSA+Cy5
CD11b/HLA-DR/IDO-1/CK	CD11b	EP1345Y	Rabbit EnVision+ HRP	Opal520
	HLA-DR	TAL.1B5	Mouse EnVision+ HRP	TSA+Cy3
	IDO-1	SP260	Rabbit EnVision+ HRP	TSA+Cy5
	Pan-Cytokeratin	AE1/AE3	N/A	Alexa488
CD4/CD8/CD25/FoxP3/Ki67/CK	CD4	4B12	Mouse EnVision+ HRP	Opal520
	CD8	C8/144B	Mouse EnVision+ HRP	Opal620
	CD25	SP176	Rabbit EnVision+ HRP	Opal570
	FoxP3	D2W8E	Rabbit EnVision+ HRP	Opal540
	Ki67	MIB-1	Mouse EnVision+ HRP	Opal650
	Pan-Cytokeratin	AE1/AE3	Mouse EnVision+ HRP	Opal690

Table S2. Clinical characteristics of biomarker cohort of patients with RCC

	Biomarker cohort, N = 26	Responders (CR, PR, mixed), N = 13	Non-responders (PD), N = 13
**Best response to ICI therapy (%)**			
**CR**	1 (3.8)	1 (7.7)	0 (0.0)
**PR**	10 (38.5)	11 (76.9)	0 (0.0)
**PD**	13 (50.0)	0 (0.0)	13 (100.0)
**Mixed**	2 (7.7)	2 (15.4)	0 (0.0)
**Median age at initiation of ICI (range), yr**	61 (44-85)	62 (47-79)	60 (44-85)
**Sex (%)**			
**Male**	20 (76.9)	9 (69.2)	11 (84.6)
**Female**	6 (23.1)	4 (30.8)	2 (15.4)
**Stage at diagnosis (%)†**			
**I**	3 (16.7)	2 (22.2)	1 (11.1)
**II**	1 (5.6)	1 (11.1)	0 (0.0)
**III**	5 (27.8)	2 (22.2)	3 (33.3)
**IV**	9 (50.0)	4 (44.4)	5 (55.6)
**Pathology†**			
**Clear cell**	17 (88.9)	8 (88.9)	8 (88.9)
**Papillary**	1 (5.6)	0 (0.0)	1 (11.1)
**Sarcomatoid**	1 (5.6)	1 (11.1)	0 (0.0)
**IMDC risk group (%)†**			
**Favorable**	3 (16.7)	2 (22.2)	1 (11.1)
**Intermediate**	9 (50.0)	6 (66.7)	3 (33.3)
**Poor**	6 (33.3)	1 (11.1)	5 (55.6)
**Previous therapies (%)†**			
**Nephrectomy**	17 (94.4)	8 (88.9)	9 (100.0)
**Radiation**	10 (55.6)	5 (55.6)	5 (55.6)
**Anti-angiogenic agent**	16 (88.9)	8 (88.9)	8 (88.9)
**mTOR inhibitor**	7 (38.9)	4 (44.4)	4 (44.4)
**High-dose IL-2**	6 (33.3)	2 (22.2)	3 (33.3)
**ICI agent (%)**			
**Nivolumab**	25 (96.2)	12 (92.3)	13 (100.0)
**Atezolizumab**	1 (3.8)	1 (7.7)	0 (0.0)
**ICI line of therapy (%)†**			
**First-line**	1 (5.6)	1 (11.1)	0 (0.0)
**Second-line**	3 (16.7)	1 (11.1)	2 (22.2)
**Third-line**	8 (44.4)	4 (44.4)	4 (44.4)
**Fourth-line+**	6 (33.3)	3 (33.3)	3 (33.3)
**Median duration of ICI therapy (range), days**	123 (27-1637)	448 (27-1637)***	73 (28-238)***
**Median survival (95% CI), months**			
**PFS**	4.03 (2.86-13.4)	13.4 (9.01-not reached)[###]	2.76 (2.33-not reached)[###]
**OS†**	22.4 (12.4-not reached)	Not reached (25.1-not reached)[#]	10.6 (5.95-not reached)[#]
**irAE while on ICI therapy (%)†**	5 (27.8)	5 (55.6)	0 (0.0)

†Clinical characteristic available only for subset of patients
***p<0.001, two-tailed Mann-Whitney U test
[#]p<0.05, [###]p<0.001, log-rank test
Abbreviations: CR, complete response; PR, partial response; PD, progression of disease; ICI, immune checkpoint inhibitor; IMDC: International Metastatic RCC Database Consortium; irAE, immune-related adverse event; CI, confidence interval; PFS, progression-free survival

Table S3. irAEs during ICI therapy in primary patient cohort

	Primary cohort, N = 94	Responders, N = 38	Non-responders, N = 56
**irAE type (%)**			
**All**	35 (37.2)	19 (50.0)*	16 (28.6)*
**Thyroid dysfunction**	12 (12.8)	6 (15.8)	6 (10.7)
**Dermatologic**	11 (11.7)	7 (18.4)	4 (7.1)
**Pneumonitis**	7 (7.4)	3 (7.9)	4 (7.1)
**Arthritis**	5 (5.3)	4 (10.5)	1 (1.8)
**Hepatitis**	5 (5.3)	5 (13.2)	0 (0.0)
**Colitis**	4 (4.3)	2 (5.3)	2 (3.6)
**Encephalitis**	3 (3.2)	2 (5.3)	1 (1.8)
**Nephritis**	2 (2.1)	2 (5.3)	0 (0.0)
**Hematologic**	2 (2.1)	1 (2.6)	1 (1.8)
**Myalgia**	2 (2.1)	1 (2.6)	1 (1.8)
**Hypophysitis**	1 (1.1)	1 (2.6)	0 (0.0)
**Diabetes mellitus**	1 (1.1)	1 (2.6)	0 (0.0)
**Adrenal insufficiency**	1 (1.1)	1 (2.6)	0 (0.0)
**Neuropathy**	1 (1.1)	1 (2.6)	0 (0.0)
**Arrhythmia**	1 (1.1)	1 (2.6)	0 (0.0)
**Other**	1 (1.1)	0 (0.0)	1 (1.8)
**Patients with irAE**	**n = 35**	**n = 19**	**n = 16**
**irAE management (%)**			
**Immunosuppression**	18 (51.4)	10 (52.6)	8 (50.0)
**Oral steroids**	17 (48.6)	10 (52.6)	7 (43.8)
**Intravenous steroids**	6 (17.1)	2 (10.5)	4 (25.0)
**Highest grade of irAE (%)**			
**Grade 1**	11 (31.4)	6 (31.6)	5 (31.3)
**Grade 2**	16 (45.7)	8 (42.1)	8 (50.0)
**Grade 3**	7 (20.0)	4 (21.1)	3 (18.8)
**Grade 4**	1 (2.9)	1 (5.3)	0 (0.0)

*$p<0.05$, OR = 2.50 (95% CI = 1.057 – 5.911), chi-square test
Abbreviations: irAE, immune-related adverse event

Table S4. Sites of metastasis prior to initiation of ICI in primary patient cohort

	Primary cohort, N = 94	Responders, N = 38	Non-responders, N = 56
**Sites of metastasis (%)**			
**All**	90 (95.7)	36 (94.7)	54 (96.4)
**Visceral**	84 (89.4)	33 (86.8)	51 (91.1)
**Lung**	67 (71.3)	29 (76.3)	38 (67.9)
**Lymph node**	57 (60.1)	23 (60.5)	34 (60.7)
**Bone**	36 (38.3)	14 (36.8)	22 (39.3)
**Adrenal**	30 (31.9)	12 (31.6)	18 (32.1)
**Liver**	28 (29.8)	8 (21.1)	20 (35.7)
**Soft tissue**	22 (23.4)	7 (18.4)	15 (26.8)
**Pancreas**	17 (18.1)	3 (7.9)*	14 (25.0)*
**Brain**	12 (12.8)	4 (10.5)	8 (14.3)
**Gastrointestinal**	5 (5.3)	2 (5.3)	3 (5.4)
**Other**	6 (6.4)	1 (2.6)	5 (8.9)

*p<0.05, OR = 0.257 (95% CI = 0.0683 – 0.968), chi-square test

Table S5. Selected differentially expressed pathways from gene set variation analysis

Pathway	Pathway type	Log_2(ratio)	P-value
GO_KETOSTEROID_MONOOXYGENASE_ACTIVITY	Metabolism	-0.03933	0.00019
GO_BILE_ACID_BINDING	Metabolism	0.007758	0.000194
GSE6674_UNSTIM_VS_CPG_STIM_BCELL_DN	Immune	-0.04345	0.001921
GSE15659_CD45RA_NEG_CD4_TCELL_VS_ACTIVATED_TREG_UP	Immune	0.007549	0.001928
GO_COLLAGEN_BINDING_INVOLVED_IN_CELL_MATRIX_ADHESION	Metabolism	-0.02938	0.002564
GSE32423_CTRL_VS_IL4_MEMORY_CD8_TCELL_UP	Immune	0.003767	0.003148
GO_NEGATIVE_REGULATION_OF_SYNAPTIC_VESICLE_TRANSPORT	Metabolism	0.032617	0.003375
REACTOME_GLYCOGEN_BREAKDOWN_GLYCOGENOLYSIS	Metabolism	-0.06214	0.003438
TONKS_TARGETS_OF_RUNX1_RUNX1T1_FUSION_GRANULOCYTE_UP	Immune	0.000645	0.003607
MORF_IFNA1	Immune	0.018098	0.003761
GO_MEDIUM_CHAIN_FATTY_ACID_COA_LIGASE_ACTIVITY	Metabolism	-0.00155	0.004061
GSE21380_NON_TFH_VS_GERMINAL_CENTER_TFH_CD4_TCELL_UP	Immune	0.011311	0.004099
GSE3982_CTRL_VS_LPS_1H_NEUTROPHIL_DN	Immune	-0.01864	0.004152
GO_PYRUVATE_TRANSMEMBRANE_TRANSPORT	Metabolism	-0.01757	0.004259
GSE17974_IL4_AND_ANTI_IL12_VS_UNTREATED_4H_ACT_CD4_TCELL_UP	Immune	-0.03967	0.004449
GSE5589_LPS_VS_LPS_AND_IL10_STIM_MACROPHAGE_180MIN_UP	Immune	-0.03487	0.005379
GO_ANTIGEN_PROCESSING_AND_PRESENTATION_OF_LIPID_ANTIGEN_VIA_MHC_CLASS_IB	Immune	0.043893	0.00661
REACTOME_SYNTHESIS_OF_BILE_ACIDS_AND_BILE_SALTS_VIA_24_HYDROXYCHOLESTEROL	Metabolism	-0.00361	0.006839
GO_REGULATION_OF_DEFENSE_RESPONSE_TO_VIRUS	Immune	-0.03198	0.008726
GSE26928_EFF_MEM_VS_CENTR_MEM_CD4_TCELL_DN	Immune	-0.01357	0.00939
GO_POSITIVE_REGULATION_OF_DEFENSE_RESPONSE_TO_VIRUS_BY_HOST	Immune	-0.02478	0.009429
GSE15659_NONSUPPRESSIVE_TCELL_VS_ACTIVATED_TREG_UP	Immune	0.017029	0.009708
